# Customer Experience and Satisfaction in Private Insurance Web Areas

**DOI:** 10.3389/fpsyg.2020.581659

**Published:** 2020-10-09

**Authors:** M. Dolores Méndez-Aparicio, Ana Jiménez-Zarco, Alicia Izquierdo-Yusta, Juan Jose Blazquez-Resino

**Affiliations:** ^1^Economics and Business Faculty, Open University of Catalonia, Barcelona, Spain; ^2^Department of Economics and Business Administration, University of Burgos, Burgos, Spain; ^3^Deparrment of Business Administration, University of Castilla-La Mancha, Ciudad Real, Spain

**Keywords:** customer digital experience, customer satisfaction, quality web, customer expectations, private customer web areas, effect WOW, insurance field, co-creation

## Abstract

Digital transformation has allowed to offer additional services—which complement the main product—both in terms of use, emotional, and relationship terms. Focusing on a traditionally rational insurance customer offering a value that explores the customer’s emotions, from co-creating with the user, allows brand differentiation. Given this idea, this document has three purposes. First is identify the true role of expectations and the perceived quality of the customer’s digital experience. Second is to identify the relationship between customer experience and satisfaction gained in private insurance web areas. Third is to identify the most valued digital attributes by the user. A sample of 4,178 customers registered was analyzed using the partial least-squares technique. The model is highly predictive to customer experience and evidence the important relationship between the WOW effect and satisfaction, as well as the weak but double role that expectations play on insurance digital self-service. The model demonstrates that expectations are only relevant before web consumption, because during the process it is the perceived digital quality, in particular the usefulness, information, and technology, that offers a true customer experience. This article offers high academic value because it more accurately defines the determinants of the digital insurance customer experience and its effect on customer satisfaction in digital services. While expectations influence attitude before service, this research reveals that perceived digital quality is what delivers a true customer experience. Strategically, the implications are immediate in the field of business as it shows the importance of co-creation in digital design, not only because of the significant savings in implementation costs but also because it guarantees a greater experience, essential in the loyalty of its customers. This ensures the sustainable growth of the company.

## Highlights

- The customer experience is predictable from customer expectations and the quality offered in insurance digital service.
- The expectations are relevant before web consumption, but during the process it is the perceived digital quality that offers a true insurance customer digital experience.
- Customer expectations influence on the perceived satisfaction indirectly, through the mediation of the insurance customer digital experience.
- The customer experience of the result of the insurance digital service impacts directly on their satisfaction with that result.
- In a digital self-service, information is the most valued attribute, which makes an agent unnecessary.
- In a virtual environment, the quality of the platform is highly valued, specifically the multi-device adaptation, speed, and ease of use.

## Introduction

Since the beginning of the 21st century, there have been multiple economic analysts who point to the relevance of services as the ability to add value to a brand (Vargo and Lusch, 2008). This feature allows companies to develop a differential competitive strategy (Ali et al., 2018), based on an innovative service offering (VI DEC Congress, 2019) offered in a unique (Blut et al., 2014) and customized way (Bilgihan et al., 2015). This new customer centricity vision (Kohli et al., 2019) implies a great knowledge of their behavior and needs (De Mooij, 2019), including affective ones (The Human Brand Report, Grass Roots, 2018). In this new interconnected socioeconomic context, it reveals that the role of consumers has changed substantially and consumer participation has become key to the development of products and services of mutual value as a source of innovative ideas and brand value (Martínez-Cañas et al., 2016). Research reveals that personalization, excellence in supply and service, and speed of delivery are critical in consumers’ value perception (Deloitte, 2017). In this way, digital channels offer companies a great opportunity to create functional value to consumers but also emotional value that as a differential tool (Zhu and Gao, 2019) can consolidate company–consumer relationship (Karjaluoto et al., 2012) since they allow the implementation of a space in which the relationship is different, even extraordinary (Carù and Cova, 2013), based on the generation of experiences and co-creation of solutions (García Haro, 2018).

Although digital reality is a fact (PWC, 2017), it is not easy to implement value self-services in all sectors and consolidate the adoption and dissemination of the web channel among customers (Nicoletti, 2016). In the case of insurance, the provision of services concerns the possibility of risk of human life, and/or the most precious assets of customers made an object of insurance (García, 2010), and where confidence that the company will meet one’s needs is the key (Capgemini, 2020). This makes the customer’s perception of this product high risk and seeks non-virtual channels (Minsait, Asociación para el Desarrollo de la Experiencia de Cliente (DEC), 2019) as a means of relationship with insurers. Guaranteeing this trust from a digital service is difficult since the private area must satisfy two profiles of well-differentiated behaviors: (1) the rational profile that prevails when contracting the insurance, where the information (Chang et al., 2014) and transaction security minimize the perceived risk (Currás-Pérez and Sánchez-García, 2012; Thakur and Srivastava, 2014) and (2) the emotional profile experienced when requesting the contracted service: reporting a mishap in the customer’s assets including the death of loved ones. In these cases, the moment of the client’s truth (IZO, 2019) on the digital channel must behave with sufficient quality and warmth (Grass Roots, 2018) that it would have received from an agent (Palm, 2016).

In short, this additional service provided by the digital channel, added to the main service, increases the value of other intangibles (dominant service logic, Vargo and Lusch, 2014) and therefore allows not only a brand experience through direct and continuous contact, but also provides other high-value elements such as effectiveness, efficiency, support and other useful services (Inbound Marketing, InboundCycle, 2020).

Despite the divergence between sectors, it is important to highlight that what has unanimously changed is the way in which consumers approach the sector and where the service is resolved in real time in a simple way (EIOPA, 2017). In the case of the insurance field, the client cannot compare the digital experience with the immediate competitors, but it will do so with the sectors with which it is digitally related (Amezua, 2019). Moreover, as the author says, social change is parallel to technology “so that adaptation to new technologies is essential to avoid the obsolescence of company services” (Amezua, 2019).

However, despite the social changes and the relevance of the digital customer experience on trust in the insurance brands (Han and Hyun, 2015) and loyalty in the consumer (Ali et al., 2018), in a sector that is 8.92 of the world GDP (OECD, 2020), the authors Pérez-Rave and Muñoz-Giraldo (2014) warn of insufficient research on the quality of service from an attitudinal perspective and studies of models of complex behaviors, where multiple variables interact.

But what is customer experience? The term “customer experience” was introduced by Holbrook and Hirschman (1982), but it was not until 1999 that it first appeared in academic literature (Pine and Gilmore, 1999). The ability to simultaneously meet functional and pleasurable needs (Tamborini et al., 2011), through surprising and memorable encounters (Ali et al., 2014), allows the creation of unique experiences (Blut et al., 2014) of a cognitive, social, affective, and physical nature between the client and the company (Vargo and Lusch, 2014).

The challenge for insurance companies is to turn digital interaction into an added value for the customer and not simply meet their needs in a way that allows them to increase their brand experience (Wanick et al., 2017), increase their reputation (De Quevedo et al., 2005), and enhance channel loyalty (Chen and Phou, 2013). New values are incorporated into the service (cite Anne’s book), beyond intrinsic functional value, such as (1) Emotional Value or capacity for well-being, emotion, and happiness in digital interaction (Lemon and Verhoef, 2016); (2) Social Value, where the client improves his position and his own identity by adopting technology (Bilgihan et al., 2016); and (3) Epistemic Value: it relates to the sense of adventure and the satisfaction of obtaining a knowledge that takes away his curiosity (related to flow perception, Nakamura and Csikszentmihalyi, 2014). Therefore, it is necessary to know the mechanism through which the digital service allows the simultaneous customer to (1) achieve the functional need that led them to use the service and (2) obtain a personal, unique, and an exclusive experience, in such a way as to produce a high degree of customer satisfaction. In this process, the quality of the web service is key to obtaining the desired result, in accordance with the initial expectations of the client (Michalco et al., 2015) and make evaluations accordingly (Kujala et al., 2017). Although the co-creation process is a common marketing practice (García Haro, 2018), in the virtual field the lack of possibility of customization of private web areas is notorious. In this process, it is not only necessary to listen to the needs of the client, but, as García Haro (2018) indicates, also necessary to incorporate the virtual consumer in the decision processes that improve the experience, such as the digital design of the services and what attributes they should possess, so that they respond to the expectations of the users of those private web areas. This requires innovation in knowledge, relationship models, new technologies, and services to provide new experiences (Marketing Science Institute, 2016).

In this socioeconomic and technological context described, in deep current change, this research sets the objective of investigating the antecedents and consequences of the digital customer experience in the insurance sector. It is intended to confirm whether the client’s behavior model presents differences compared to the reference framework consulted due to the different bias that differentiates it from other services. On the one hand, the one that provides the adoption of technology in a traditionally non-digital sector (Thakur and Srivastava, 2014; Minsait, Asociación para el Desarrollo de la Experiencia de Cliente (DEC), 2019; Capgemini, 2020). Second is the risk due to the very nature of its coverage (García, 2010). In this new business strategy focused on the customer experience (Comisión nacional de los Mercados y la Competencia [CNMC], 2019), where users expect quality services, congruent with the value of the brand (Rather and Camilleri, 2019), it seems necessary to delve into the sector and relate consumer expectations and quality of service as a background to the experience. Research that has not been identified in the reference literature on the digital attributes is considered. Although it would be necessary to reel how the customer experience is shaped from cognitive, affective, and behavioral dimensions (Rather, 2020), this research raises three objectives specific about the digital customer experience: (a) the objective of verifying whether the user’s attitude is relevant to user satisfaction from the perceived experience, that is, identify the true role of expectations and the perceived quality of the customer’s digital experience, (b) identify the relationship between customer experience and satisfaction gained in private insurance web areas, and (c) identify the most valued digital attributes by the user. For it, this article proposes an analysis of these private customer web areas, where it is possible to measure and analyze customer behavior through a relational model of customer experience. To do this, it begins by analyzing the relationship between the following four variables: *expectations*, *quality*, *customer experience*, and *satisfaction*.

The results statistically confirmed the robustness of the model, which allows the customer experience to be considered as predictive based on the proposed determinants and, therefore, to formulate implications/conclusions of great interest to the scientific community in the knowledge of the behavior of the digital consumer. However, the business implications are also noteworthy as they promote in the business community the consolidation of Customer Experience programs and offer immersive digital services, essential for the sustainability of companies given the influence that the customer experience has on satisfaction and this on trust (Han and Hyun, 2015) and loyalty to the company (Yoo et al., 2013) and the adoption of the new communication channels (Chen and Phou, 2013). Knowing the best communication strategy with one’s client by new channels can bring considerable savings in companies in their digital transformation (Comisión nacional de los Mercados y la Competencia [CNMC], 2019) in order to optimize million-dollar investments in advertising, technology, and better information systems (SI).

## Theoretical Framework and Hypothesis

According to the iabSpain (2018), the new customer profile is that of the connected individual. Intensive use of digital channels enables this individual to maintain a continuous, direct, and interactive relationship with the company. This demands a highly personalized product and service offering as well as immediacy and agility in the delivery of value (Deloitte, 2017).

This new type of relationship enables customers to formulate expectations in relation to what they expect to receive from the digital service, on which they will evaluate the results obtained in terms of experience and satisfaction according to the quality presented by the web service.

### Knowledge of the Expectations of a Digital Service

Expectations are the subject of continuous research in the marketing environment. Their importance in the consumer decision processes (Guadarrama Tavira and Rosales Estrada, 2015) makes effective expectation management a strategic objective for companies (Noe et al., 2017).

The many definitions offered on this concept demonstrate that expectations are formed in the mind of the individual and are therefore subjective in nature (Pelegrín-Borondo et al., 2016), subject to the individual’s hope (Real Academia Española, 2019), belief, or idea (Warren, 2018) that something might happen or take effect in a certain situation (Peralta Montecinos, 2015). Expectations are also the benchmark by which the individual compares (Oliver, 2014) and evaluates (Rodríguez del Bosque and San Martín, 2008) the result obtained. Expectations, therefore, set the threshold for the minimum accepted by the individual.

It should be noted that expectations evolve over time according to changes to the context in which the relationship takes place (Pelegrín-Borondo et al., 2016). As the individual gains experience, however, their expectations become more stable and are transformed into a generalized belief held by the individual in relation to what can be received (Meyer and Harris, 2012).

Due to their psychological nature, expectations clearly play an important part in the development of an individual’s emotional attachment to a brand (Pelegrín-Borondo et al., 2017). The analysis and management of expectations therefore offers companies a strategic opportunity during the consumer’s decision-making process (Gómez Borja, 2014), thanks to the creation of channels through which companies can relate to their customers in a technological context (De Keyser and Lariviere, 2014; Wang et al., 2016). Specifically, in relation to a digital service such as private customer web areas, the individual establishes expectations in terms of (1) the service’s ability to respond to a need (competence or capacity to resolve a problem) and (2) the way in which this response is offered (digital procedure or interaction). Also, the technological component that sustains this process is as fundamental to what the individual expects as to what they receive (Swaid and Wigand, 2012; Chang et al., 2014; De Keyser and Lariviere, 2014; Wang et al., 2016).

Numerous studies recognize the role of expectations in the experience and satisfaction the customer receives (Cheung et al., 2015; Huang et al., 2015; Ali et al., 2016). The fact that experience and satisfaction are closely related is worthy of mention. However, whereas experience is how the customer feels during and after the interaction with the service offered by the brand, satisfaction is the direct consequence of the result of the process (San Martín Gutiérrez et al., 2008). In the case of digital channels, it can be said that the customer’s expectations of how the technology enables the service will determine the experience (Kujala et al., 2017). Furthermore, a customer’s expectations of the technology’s capacity to offer the service will affect the satisfaction obtained (Bilgihan et al., 2015).

The degree of disconfirmation between the expectation and the result obtained will determine the experience obtained by the customer (Oliver, 2014; Michalco et al., 2015). Therefore, the greater the difference between what is expected and what is received, the greater the experience (positive or negative) obtained by the customer. Experience and disconfirmation function in the same direction (De Santiago, 2015; Pons, 2015; Action Coach, 2018; Círculo Marketing, 2018; Asociación para el Desarrollo de la Experiencia de Cliente [DEC], 2018; Deloitte, 2018; MdS, 2018). This discrepancy between what is expected and what is received becomes extremely important for individuals in situations in which they have a high degree of engagement in the process either because of the importance they give to the process (Peralta Montecinos, 2015) or because of one’s expectations about the brand (Michalco et al., 2015), because this discrepancy surprises and alarms them (Geers et al., 2009). Where the level of engagement is low, therefore, the relationship between the expectation and the experience will be of little significance (Alba and Williams, 2013), though it should be understood that either the individual will modify or readjust their expectations or the experience will be adjusted to minimize the degree of disconfirmation. As can be deduced, for companies it is complex to provide experience and satisfaction from the fulfillment of expectations, given its subjective and therefore diverse nature. Thus, taking into consideration *contrast theory*, the customer will distort their expectations to fit the experience, thus enabling them to justify their choice (Pelegrín Borondo, 2013). On the other hand, according to *assimilation or dissonance theory*, the opposite may occur, with the consumer adapting the experience to the expectation in such a way as to produce a distortion of the reality (Raita and Oulasvirta, 2011; Oliver, 2014; Michalco et al., 2015). For this reason, this research considers how expectations influence before the process and after the process, that is, what you expected from the service and what impact its contrasts with what you received have on it.

Expectations are also considered a determinant of customer satisfaction. The individual will be satisfied to the extent that the service they receive meets or exceeds what they expected (Ali et al., 2016), confirming their expectations in terms of the capacity of the service to respond to a need or resolve a problem (Oghuma et al., 2016). Contrary to what occurs with customer experience, when expectations are transformed into reality, the individual acquires a feeling of control over the medium and therefore experiences satisfaction, having avoided loss or harm (Guo et al., 2016; Zehrer and Raich, 2016).

In the current socioeconomic context, technology plays a decisive role in the relationship of expectations and perception of service by the user (Mallaina García, 2017; Molina et al., 2017; Rey-García et al., 2017). This research contextualizes the true role of customer expectations in the digital experience to determine how important the digital channel’s impact on online customer and business interaction is. While the theoretical reference framework indicates that expectations also influence during the process and even beyond post-purchase (De Keyser and Lariviere, 2014), the model allows to contextualize the customer’s digital expectations and conclude which, while relevant to the consumer at the time of consumption before, perceived quality is the variable that most influences the experience during and after the digital transaction. Moreover, this experience directly influences satisfaction, regardless of the expectations formed before the consumption of the service (Ali et al., 2016).

On the other hand, the results of the research demonstrate the acquired importance of the web channel to the user in such a way that it becomes the object of specific future expectations in relation to (1) the operation of the platform, (2) the quality of the information that accompanies the process, (3) the technological implications of the service, and (4) the new customer–company relationship that arises thanks to the new channel (Zavareh et al., 2012).

Based on the arguments posited, the following hypotheses are formulated:

**H1***: The customer’s expectations of the result of the digital service directly impact on the customer’s experience with that result.*

**H2***: The customer’s expectations of the result of the digital service directly impact on the customer’s satisfaction with that result.*

### Perceived Quality of the Digital Service

Service quality becomes increasingly important, since it is used as an additional element that enriches the offering and increases the value received by the customer (Guo et al., 2019). In particular, private customer web areas are presented as a medium for promotion of a closer, more interactive, and personalized customer–company relationship (Chang et al., 2014). This renders service quality a determinant in the customer’s experience (Ali et al., 2016).

However, despite the interest aroused, the multidimensional nature of the concept means there is no consensus as to its definition (Cruz Sánchez et al., 2018). These authors define quality as excellence or the maximum quality achievable. Cruz (2004) defines quality as “the ability of the organization to ensure that its products and services meet their customers’ implicit needs.” This definition is corroborated by ISO 9001:2015 (2016), which states that “quality is determined solely by the characteristics defined by the customer.” Worthy of note in this respect is the fact that quality is as perceived by the individual and is dependent on personal taste and individual expectations (Méndez Aparicio, 2019).

The scientific literature has identified the various dimensions considered by individuals when evaluating the quality of a service. Of particular relevance is the research conducted in the 1990s by Zeithaml, Parasuraman and Berry, who identified as many as ten elements on which customers base their perceptions and expectations of quality (Parasuraman et al., 1994; Zeithaml and Bitner, 2002) and which have been a quality standard for many years.

Despite the different characteristics of the digital environment, Alcaide Casado and Soriano Soriano (2006) conclude that the services offered in private customer web areas are not noticeably different from those provided in face-to-face services. Accordingly, researchers enumerated the following characteristics as equally necessary in a web service: *reliability*, *response capacity*, *professionalism*, *accessibility*, *courtesy*, *communication*, *credibility*, *security*, *knowledge of the customer*, and *tangible elements*. For Chang et al. (2014) and Wang et al. (2016), these attributes of quality guarantee the expected utility and are the elements upon which the customer will base their expectations. These characteristics will also condition the adoption of the digital channel as the habitual relationship with the brand (Izquierdo Yusta et al., 2011; Méndez Aparicio, 2019).

In Rather (2020), the customers will continuously evaluate the service quality during their interactions with the business. This has sparked the interest of companies in customer area technology management, who need to consolidate their digital relationship with the client. As responsible for customer satisfaction, companies must ensure success in their interaction with customers and how they feel during the process (Olarte-Pascual et al., 2016). Thus, multiple study proposals have measured the quality of digital service from multiple perspectives (Méndez Aparicio, 2019). Noteworthy is the study by Hsu et al. (2012) and Chang et al. (2014) that has allowed the identification of three determining dimensions of digital service quality: *technical quality*, *service quality*, and *information quality.* According to ISO Standard 25010 (ISO, 2019), these elements are sufficient to measure the quality of the website atmosphere (Kobusińska and Hsu, 2018; Shao et al., 2019).

This proposal is considered in this research as the most appropriate and complete, since it contemplates the three aspects of digital quality: the quality information that accompanies the user, the quality of the brand promise received, and the quality of the platform in the one that is served. However, despite being orderly and systematic, it suffers from important attributes, justified by other authors, such as security (Swaid and Wigand, 2012) and perceived risk in the service (Wai et al., 2019), essential in a virtual environment that must guarantee trust. Finally, it is also considered important to incorporate the efficiency or capacity to have the resources (services, systems, or information) necessary to achieve the desired service (Swaid and Wigand, 2012). Therefore, these aforementioned attributes will also be incorporated into the research to complete the gap detected in the frame of reference. Ultimately, this study aims to confirm whether the following digital attributes are indicators of customer experience and, therefore, can be considered as a benchmark for an excellent web atmosphere:

(a) The information must be relevant, unambiguous, concrete, sufficient, and up-to-date.
(b) The quality of the brand promise received must provide data-safe, interactive, comprehensive, and fast services.
(c) The platform on which the service is implemented must be free of navigation risks, have a multi-device adaptation, be reliable, easy to use, fast, and attractive.

It is clear that the customer’s interaction with the organization depends on technological aspects, so that the quality of the software used determines accessibility, ease of use, speed, reliability, multi-device adaptation, and security of service delivery (Huang et al., 2015). The quality of the technical component also impacts on the usability of the app or the website, its appeal, and its capacity to generate a website atmosphere that improves the customer’s experience (Michalco et al., 2015). The information that the company places at the customer’s disposal is also key to the relationship (Swaid and Wigand, 2012). Having access to accurate, up-to-date, quality information facilitates and speeds up certain processes at the same time as it promotes trust and commitment to the brand on the part of the customer (Swaid and Wigand, 2012; Venkatesh et al., 2012). Finally, service quality presents a utility component that impacts on trust in the results obtained (Hsu and Lin, 2015; Wang et al., 2016), data handling security (Thakur and Srivastava, 2014), and, above all, complete service autonomy (Chang et al., 2014).

Based on the arguments posited, the following hypothesis is formulated:

**H3:** *The quality of the digital service impacts directly on the customer’s experience.*

### Customer Experience and Its Impact on Customer Satisfaction

Customer experience and satisfaction are among current business objectives. Despite being closely related, there are differences between the two concepts (San Martín Gutiérrez et al., 2008).

Experience is how the customer feels during and after the interaction with the service offered by the brand. It is defined by Lemon and Verhoef (2016) as memorable experience, and, along the same line of reasoning, by Tokman et al. (2007) as “surpassing all expectation, whether in shortfall or excess, and resulting in full satisfaction—or dissatisfaction—and a memorable experience.” The same argument is shared by Oh et al. (2007), who point to the emotional nature of the experience, derived from “entertaining, pleasurable, memorable and striking encounters.” In this way, as indicated by Vargo and Lusch (2014), the complete nature of the interaction, during which pleasurable experience combines with the utilitarian nature of cognitive, affective, physical, and social dimensions, creates memorable personal experiences (Adhikari and Bhattacharya, 2016).

Among the primary focuses of research into customer experience have been understanding how the set of emotions, sensations, and sensory images felt by the individual are interpreted and internalized (Walls et al., 2011; Duerig et al., 2013) and stored in the memory (Sierra Diez et al., 2010; Asociación para el Desarrollo de la Experiencia de Cliente [DEC], 2017). Moreover, the fact is that, at the strategic level, companies do not only need the result of the different service encounters between the customer and the brand must be positive (The Human Brand report, Grass Roots, 2018). It is also essential for the brand to ensure the customer’s personal involvement on a sensory, emotional, cognitive, physical, and relational level (Chathoth et al., 2014; Lemon and Verhoef, 2016).

The principal effects of the experience on the customer’s behavior are another focus of interest in relation to this concept. Studies conducted by Evans et al. (2009) and Iannini (2010), among others, have demonstrated the strategic importance for companies of generating positive experiences in their customers. A positive experience establishes an emotional connection between the brand and the customer, which generates in the customer a positive response to the brand and motivates future behavior and interaction (Sierra Diez et al., 2010).

Satisfaction is confirmed as one of the primary results of experience and is defined in terms of the pleasure (Wang, 2011) and enjoyment (Ali et al., 2018) felt by the customer as a direct consequence of the result obtained from the service (Capgemini, 2020). However, it is also true that a key element of the end value provided by a service is determined by the way in which the service is offered; in other words, how the interaction between the customer and the brand was conducted (Ali et al., 2018). Thus, the inseparability of the production and consumption of the digital service conditions the result of the interaction and its capacity to deliver value and, therefore, the customer’s experience (Alcaide Casado and Soriano Soriano, 2006; Vargo and Lusch, 2014). This consideration is relevant, since in previous studies developed in the effervescence of ICT, it is common to identify experience with hedonism, vandalizing the process from a commercial and playful perspective (Oh et al., 2007). However, as the results of the research show, the client highly values seeing their service expectations exceeded, when they obtain an excellent perceived quality and utility (Tsai et al., 2011). Therefore, this conclusion obtained is not only novel but relevant to connected users and in a current post-pandemic context due to COVID-19, in which new values such as effective, efficient, and safe service are becoming the levers for the use of the digital channel in the immediate future but also in the medium and long term, as Findasense indicates in the COVID-19 study of April 2020 (Findasense, 2020).

Finally, satisfaction is considered a fundamental objective in the company’s strategy (Arango Serna et al., 2012; Hoekstra et al., 2015) because of its considerable impact on the customer’s trust in the company (Lassala Navarré et al., 2010), on repeat purchases of products and services (Currás-Pérez and Sánchez-García, 2012; Prado Román et al., 2013), on repeated use of the channel (Mendoza-Tello et al., 2018), and on recommendations to third parties (Lin and Lekhawipat, 2014; Strong View, 2014; Méndez Aparicio, 2019).

This consideration makes this research necessary as it generates knowledge to design business strategies built on the customer experience (Triantafillidou and Siomkos, 2014).

Based on the arguments posited, the following hypothesis is formulated:

**H4:** *The customer’s experience of the result of the digital service impacts directly on their satisfaction with that result.*

## Materials and Methods

To be sure to choose a robust digital sample, it was therefore necessary to select a relevant company in the insurance business whose digital presence is indisputable (El Independiente, 2018; La Vanguardia, 2019), with plenty of customer area activity (21.000 monthly accesses). Only two criteria were taken into account in extracting the representative sample: (1) contractual continuity of three months and (2) having performed at least one operation in the private customer web areas during the previous month, to ensure the customer’s familiarity with both the company and the channel. The requested online service covers any operation: contracting, claiming, or requesting insurance services. The scope includes auto, motorcycle, home, life, or savings insurance.

Once the state of the art had been reviewed, and the hypotheses and the model to be tested had been defined, in-depth interviews with experts were carried out to confirm the most appropriate indicators for the online channel and how the approach to the customer should take place.

To confirm the hypotheses formulated, 4 constructs were identified that would allow studying the underlying relationships questioned: *digital quality*, *expectations*, *experience*, and *customer satisfaction.* The corresponding reference framework was established on the proposed relationships and, in particular, in the digital context (see Table 1). Similarly, the most precise indicators that define them were defined: 4 items to measure expectations; 17 to assess the digital quality of the service; 2 for customer experience; and 5 to measure satisfaction. In total, 28 scales were incorporated into the definitive form, ratified again by experts, from the qualitative methodology proposed. The choice of an 11-point Likert scale ensured adequate variance in the responses (Bisquerra Alzina and Pérez Escoda, 2015) and enabled the subsequent treatment of the data according to groups of points obtained.

**TABLE 1:** Justification of scales used.

Name of item	Description of item	References
**Expectations of the digital channel:**		
EXPGL1	Overall expectations	Knutson et al. (1990)
EXPGL2	Expectations of information	Swaid and Wigand (2012), Chang et al. (2014), Michalco et al. (2015)
EXPGL3	Expectations of the process	Swaid and Wigand (2012), Chang et al. (2014), Michalco et al. (2015)
EXPGL4	Expectations of the system	Swaid and Wigand (2012), Chang et al. (2014), Michalco et al. (2015)
**Quality of digital information:**		
CAIN1	Important information	Yang and Jun (2002), Long and McMellon (2004), Hsu et al. (2012), Swaid and Wigand (2012), Chang et al. (2014)
CAIN2	Clear information	Loiacono et al. (2000), Yang and Jun (2002), Long and McMellon (2004), Hsu et al. (2012), Swaid and Wigand (2012)
CAIN3	Detailed information	Yang and Jun (2002), Hsu et al. (2012), Swaid and Wigand (2012), Chang et al. (2014)
CAIN4	Sufficient information	Madu and Madu (2002), Yang and Jun (2002), Hsu et al. (2012), Swaid and Wigand (2012), Chang et al. (2014)
CAIN5	Updated information	Hsu et al. (2012), Swaid and Wigand (2012), Chang et al. (2014)
CAIN6	Quality information	Liu and Arnett (2000), Waite and Harrison (2002), Yang and Jun (2002), Hsu et al. (2012),
**Digital service quality:**		
CASE1	Information on data security policy	Madu and Madu (2002), Caruana and Ewing (2006), Kassim and Asiah Abdullah (2010), Swaid and Wigand (2012), Chang et al. (2014), Thakur and Srivastava (2014)
CASE2	No support agent needed	Long and McMellon (2004), Yang et al. (2004), Swaid and Wigand (2012), Chang et al. (2014)
CASE3	Reliability: expected results	Parasuraman et al. (1985), Zeithaml and Bitner (2000), Madu and Madu (2002), Wolfinbarger and Gilly (2002), Yang and Jun (2002), Long and McMellon (2004), Yang et al. (2004), Swaid and Wigand (2012), Chang et al. (2014)
CASE4	Speed of service	Yoo and Donthu (2001), Yang and Jun (2002), Swaid and Wigand (2012), Chang et al. (2014)
CASE6	Excellent service	Liu and Arnett (2000), Madu and Madu (2002)
**Perceived quality of digital system:**		
CASI1	Data security	Liu and Arnett (2000), Zeithaml and Bitner (2000), Madu and Madu (2002), Caruana and Ewing (2006), Kassim and Asiah Abdullah (2010), Swaid and Wigand (2012)
CASI2	Multidevice adaptation	Zeithaml and Bitner (2000), Bauer et al. (2006), Swaid and Wigand (2012), Klaus (2013), Chang et al. (2014), Bilgihan et al. (2015), Huang et al. (2015)
CASI3	System availability	Swaid and Wigand (2012), Al Sokkar and Law (2013), Chang et al. (2014)
CASI4	Speed of response	Cai and Jun (2003), Long and McMellon (2004), Bauer et al. (2006), Kassim and Asiah Abdullah (2010), Chang et al. (2014)
CASI5	Attractive design	Liu and Arnett (2000), Caruana and Ewing (2006), Bauer et al. (2006), Hartmann et al. (2008), Swaid and Wigand (2012), Al Sokkar and Law (2013), Michalco et al. (2015)
CASI6	Platform quality	Cox and Dale (2001), Waite and Harrison (2002), Zavareh et al. (2012), Al Sokkar and Law (2013), Michalco et al. (2015)
CASI7	Ease of use	Loiacono et al. (2000), Zeithaml and Bitner (2000), Cox and Dale (2001), Yang and Jun (2002), Gefen et al. (2003), Long and McMellon (2004), Yang et al. (2004), Kassim and Asiah Abdullah (2010), Chang et al. (2014)
**Digital customer experience:**		
SATI1	Customer experience: Accomplishment of proposal	Knutson et al. (1990), Oliver (2014), Suarez Álvarez et al. (2007), San Martín Gutiérrez et al. (2008)
SATI2	Customer experience: Surpassing of expectations	Knutson et al. (1990), Oliver (2014), Carù and Cova (2008), Suarez Álvarez et al. (2007), San Martín Gutiérrez et al. (2008)
**Perceived satisfaction with the digital experience:**		
SAIN6	Satisfaction with information	Swaid and Wigand (2012), Chang et al. (2014)
SASE6	Satisfaction with service	Madu and Madu (2002), Swaid and Wigand (2012), Chang et al. (2014)
SASI6	Satisfaction with channel	Swaid and Wigand (2012), Chang et al. (2014)
SAUS	Satisfaction with device	Bauer et al. (2006), Klaus (2013), Bilgihan et al. (2015), Huang et al. (2015)
SAIN6B	General satisfaction	Swaid and Wigand (2012), Chang et al. (2014)

In June 2016, three mass mails were sent to the 23,223 sample candidates. The sample is indeed large to guarantee the required 95% confidence level and a sampling error of ± 1.5%. It also guarantees the behavior of more than 250,000 registered users, who carry out all kinds of operations. This is to avoid bias in the operation, age, and type of insurance that the client has. It should be added that the proposed sample size contemplated the possibility of studying segments of it. In this way, it is guaranteed that with a single fieldwork we could have enough population to study the influence of the client’s previous attitude toward the company (NPS factor). As a future line of research, also work on the experience bias is produced by the platform: url/App. As a result of this, 4,178 valid surveys were obtained online (see Table 2), in accordance with the required 95% confidence level. Participation was encouraged to increase response and interest in the research project.

**TABLE 2:** Survey technical data.

Universe	253,174 active users
Town/city/village	23,223 insured individuals with access to the services the month prior to sampling
Sample	4,178 insured individuals
Degree of trust	95%
Sample error	± 1.5%
Maximum variance supported	P = q = 0.5
Survey date	1-25 June 2016
Survey procedure	Mass mailing with link to questionnaire sent to all registered users

The descriptive analysis indicated online users with an average age of 44; the majority (30%) aged between 36 and 45, followed by groups aged 26–35 and 46–55 years. The online purchase volume was once a month, the average being 3.9 purchases per month, far higher than the Spanish national average of 3.4 recorded in 2016 (iabSpain, 2016). The computer was the platform habitually used for the consumption of services (71.9%), though the use of smartphones was already identified as a growing trend, with 18.1% of users opting for m-commerce. A total of 78% of the users regularly used the digital medium for their transactions; 79% expressed a preference for it because of the convenience of the operating hours. A total of 88.4% of the respondents had not used the App.

The digital profile obtained was fully current in 2018 (iabSpain, 2018); hence, the results were valid, providing a sample of expert users able to objectively evaluate website quality without any issues of poor adjustment to technology, producing biases with respect to perceived satisfaction.

**FIGURE 1:**
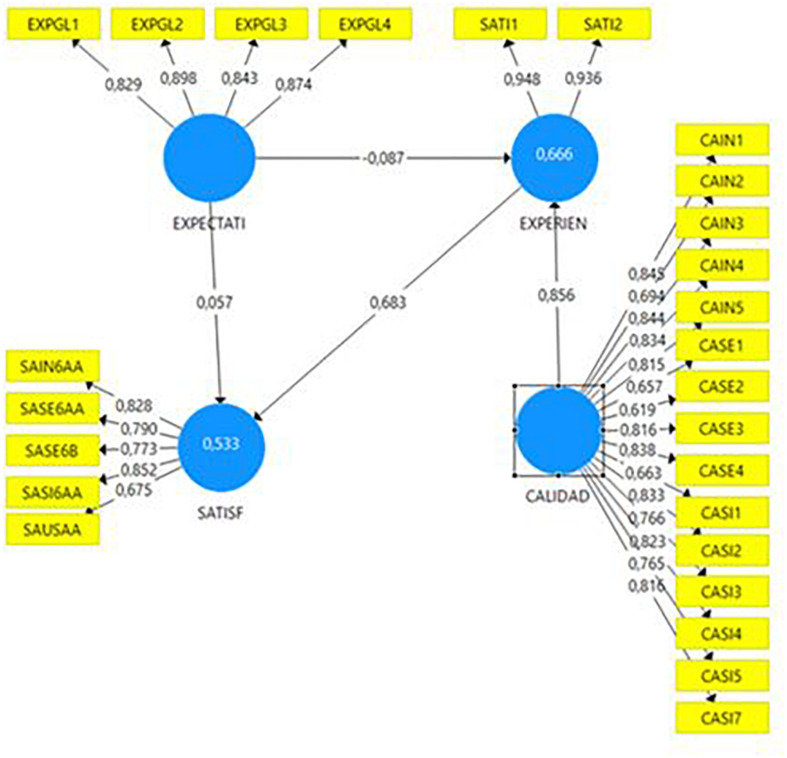
Structural model of Customer Experience.

The proposed relational model was validated using partial least squares structural equation modeling (PLS/SEM) (statistical output of SmartPLS 3, confirmed in the Figure 1) and Smart PLS 3.0 software (Ringle et al., 2015). Using a bootstrap sampling technique, the 10.000 repetitions performed (Hair et al., 2017) ensured the individual reliability of the item, the scale, the convergent validity, and the discriminant validity obtained through Cronbach’s alpha statistics, composite reliability, % of accumulated variance, the determinant of the correlation matrix, the Barlett test of sphericity, and the KMO index, as well as other important reliability statistics for PLS, such as R^2^ and the Stone-Geisser and Q2 index offered by the PLS predict. Thanks to verification, the reliability and validity of the items and constructs, as well as their predictive capacity, were confirmed, as will be seen in the results.

## Results

Partial least squares structural equation modelling uses a two-step estimation process: evaluation of the measurement model and the structural model. To analyze the measurement model, it is necessary to assess the reliability, convergent validity, and discriminant validity. As we can observe in Table 3, to ensure item validity, items with values less than 0.7 were removed (Bagozzi and Yi, 2012). The composite reliability (CR) and Cronbach’s alpha were calculated in the same way for values greater than 0.7 (Cronbach, 1951; Malhotra, 2008). The average variance extracted (AVE) was contrasted with the recommended value equal to or greater than 0.5, as indicated by Fornell and Larcker (1981). The rho_A coefficient (Dijkstra and Henseler, 2015) with a value close to 1.0 indicated the reliability of the model analyzed with PLS. The discriminant validity of the measurement model was also accepted where a given construct was different to the others (Authors’ note: It should be noted that this measurement is only applicable to constructs with reflective indicators, as is the case in this research project.) Fornell and Larcker (1981) established the need for the variance shared between a given variable and its attributes to be greater than that shared with the other variables of the proposed model, as we can observe in Table 4. The diagonal of Table 4 shows the value of the square root of the AVE for the corresponding construct, which met the criterion that the correlations between constructs must be less than the square root of the AVE.

**TABLE 3:** Reliability and validity of item and construct.

Dimension	Indicator	Value > 0.6	Cronbach’s alpha; ≥ 0.70.8	Composite reliability ≥0.6	AVE >0.5	rho_A > 0.6	Q^2^> 0.15	R^2^≤1
Expectations	EXPGL1	0.831	0.884	0.920	0.742	0.887	0.203	0.290
	EXPGL2	0.898						
	EXPGL3	0.842						
	EXPGL4	0.872						
Experience process	SATI1	0.948	0.873	0.940	0.887	0.879	0.564	0.666
.	SATI2	0.936						
Perceived quality	CAIN1	0.845	0.953	0.958	0.607	0.958		
	CAIN2	0.694						
	CAIN3	0.844						
	CAIN4	0.834						
	CAIN5	0.815						
	CAIN6	Eliminated						
	CASE1	0.657						
	CASE2	0.619						
	CASE3	0.816						
	CASE4	0.838						
	CASE6	Eliminated						
	CASI1	0.663						
	CASI2	0.833						
	CASI3	0.766						
	CASI4	0.823						
	CASI5	0.765						
	CASI6	Eliminated						
.	CASI7	0.816						
Perceived satisfaction	SAIN6AA	0.834	0.851	0.890	0.621	0.892	0.271	0.523
	SASE6AA	0.789						
	SASI6AA	0.855						
	SAUSSA	0.687						
.	SASE6B	0.762						

**TABLE 4:** Measurement instrument: discriminant validity.

	Expectations	Experience process	Quality	Satisfaction
Expectations	0.861			
Experience process	0.336	0.942		
Quality	0.495	0.813	0.779	
Satisfaction	0.345	0.715	0.73	0.788

As we can observe in Table 5, the predictive capacity of the constructs is also confirmed. Using the Stone–Geisser test, the values obtained for Q2 in customer experience of 0.585, Q^2^ by predictive PLS of 0.665, exceed by far the theoretical threshold of 0.35 for highly predictive constructs (García Haro, 2018). The same happens with the satisfaction construct, with values of 0.282 and 0.522, respectively. Regarding the values of R^2^ (maximizing the explained variance of the dependent variables, Chin and Newsted, 1999), The values obtained for customer experience (0.666) and satisfaction (0.533), higher than 0.5, confirm the robustness of both constructs.

**TABLE 5:** Predictive relevance test: R^2^ (maximization of the explained variance of the dependent variables); Q^2^ or Stone-Geisser test and Q^2^ offered by predictive PLS.

	R^2^	Q^2^ (= 1-SSE/SSO)	Q^2^ offered by predictive PLS
Customer Experience	0,666	0,585	0,665
Satisfaction	0,533	0,282	0,522

**FIGURE 2:**
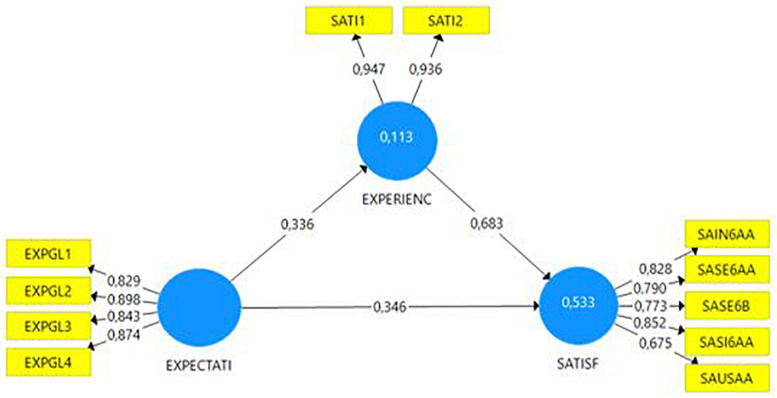
Study of total effect of Expectations on Satisfaction during the Customer Experience.

Finally, the measurement model is analyzed. As we can observe from Table 6, all the proposed hypotheses were accepted. Coefficients with a 99% confidence level were accepted, using Student’s *t*-test values greater (in absolute value) than 2.58, and where a *p*-value of less than 0.01 was satisfied. The path coefficient values (β), or standardized regression coefficients, indicated a strong relationship between both digital customer quality and experience (β = 0.856; *p* < 0.01; **H3**) and customer experience and satisfaction (β = 0.675; *p* < 0.01; **H4**). The hypothesis regarding the influence of expectations on customer experience was accepted despite the negative value of the path coefficient, which indicated a negative correlation between two variables (β = −0.087; *p* < 0.01; **H1**). Given the robustness obtained from the *p*-value and bootstrap-*t* statistics for both hypotheses (Table 6), the relationship between expectations and satisfaction (β = 0,059; *p* < 0.01; **H2**) was also accepted, despite the low value of the path coefficient (β). Moreover, **H1** and **H2** were admitted on the basis of the conclusions obtained from studying the measurement of customer experience according to expectations and satisfaction (statistical output of SmartPLS 3, confirmed in Figure 2), whereby the path coefficient (β) values for **H1** (β = 0.336; *p* < 0.01; **H1**) and **H2** (β = 0,346; *p* < 0,01; **H2**) are highly significant, as we will argue below.

**TABLE 6:** Hypothesis contrast analysis.

Hypothesis	Structural relationship	path coefficient (β)	Bootstrap-*t* value	Hypothesis contrast
H1	Expectations → Customer experience	− 0.087***	7.065	ACCEPTED
H2	Expectations → Satisfaction process	0.059***	8.350	ACCEPTED
H3	Perceived quality → Customer experience	0.856***	91.094	ACCEPTED
H4	Customer experience → Satisfaction	0.675***	69.331	ACCEPTED

***p < 0.01.

## Conclusion

The proposed client–company relationship model investigates the underlying relationship between user expectations in the face of virtual interaction, digital quality, and experience and perceived satisfaction in private insurance areas. As can be seen in the results, the model is robust and highly conclusive to predict customer experience in private insurance web areas. Whereas the usual theoretical framework places the primary focus of interest on attracting new users, this analysis enables a different perspective which stresses the importance of research into the omni-channel experience of registered users.

There are extensive and continual examples in the literature which directly link quality to customer satisfaction (Hoekstra et al., 2015; Héctor San Martín et al., 2019) and which even include service experience attributes among their value scales (Al Sokkar and Law, 2013; Bilgihan et al., 2015). However, it has become necessary to include the perspective of the event or service that produces it (Walls et al., 2011; Lourido Gómez and Otero Neira, 2016; Olarte-Pascual et al., 2016; Pelegrín-Borondo et al., 2017), applying a behavioral model to independently study how quality is experienced differently by the customer at different stages during the process: what they expected before experiencing the service (Basfirinci and Mitra, 2015; Kujala et al., 2017) and whether their expectations were fulfilled (Hall, 2012; Oliver, 2014), or even surpassed, in absolute terms (Michalco et al., 2015; Schmitt et al., 2015). The impact of the “wow” effect on customer satisfaction is amply demonstrated in the fourth hypothesis, **H4**. This conclusion is important for business growth management strategy in terms of generating both customer loyalty (Yoo et al., 2013; Lin and Lekhawipat, 2014; Ali et al., 2018) and consolidation of the use of the digital channel used (Brown et al., 2014).

With respect to quality, and as we were able to observe by comparing Figures 1, 2, a comprehensive study of the model enables us to draw five important conclusions:

(1) The importance of digital quality in customer experience is corroborated in **H3**, to the point of significantly altering the value of the path coefficient (β) in terms of what the customer expects of the service in the absence of this construct. This situation confirms that expectations are relevant in a hypothetical model of the customer–company relationship (Izquierdo-Yusta et al., 2015; Wang et al., 2016), but it is the perceived quality that should characterize an area of clients (Al-Debei et al., 2015; Hoekstra et al., 2015). Affirmation, albeit intuitive, affords insurance companies the invaluable opportunity to achieve a definitive customer experience, by ensuring high digital quality, in accordance with the company’s reputation (De Quevedo et al., 2005), technological innovation (Jiménez-Zarco et al., 2019), previous experience (Thakur and Srivastava, 2014), and third-party recommendations (Roy et al., 2013; Esteban et al., 2014; Méndez-Aparicio et al., 2017), in a novel and surprising, unpredictable way (Ngo and O’Cass, 2013; Skålén et al., 2015). In this process, co-creation with the virtual user not only provides essential information for web designers about what the ideal digital service should look like (Romero and Molina, 2011) and what needs it must meet (Hoyer et al., 2010), which improves its implementation (Kristensson et al., 2008), but generates creative ideas (Ramaswamy and Chopra, 2014; Martínez-Cañas et al., 2016), highly valued by the client participating in the process (Kristensson et al., 2008; Martínez-Cañas et al., 2016) and strengthening and consolidating the relationship between company and customer (García Haro, 2018).
(2) The importance of information as the most valued digital quality attribute in private insurance areas. According to ISO Standard 25010, the relative importance of eighteen characteristics must be considered when designing and consolidating a better, more highly regarded web channel. The first and most visible characteristic is the contribution to the construct of the information architecture, which must be *relevant*, *detailed*, *current*, *precise*, and *sufficient* (Chang et al., 2014), in accordance with the customer’s expectations (factor 0.898). The importance of information is understandable within the concept of digital self-service in insurance web areas, and the model confirms the client’s positive assessment of experiencing services where the intervention of a support agent is not necessary (Palm, 2016). This doubly satisfies the user: first, the private area responds to their new digital habits, and second, the customer feels accompanied in the service of a product called an *experience product* (Gómez Borja, 2014).
(3) The implementation of the platform is relevant in the new behavioral economy (Comisión nacional de los Mercados y la Competencia [CNMC], 2019). Next in order of importance are *speed* (Swaid and Wigand, 2012), *multidevice adaptation* (Huang et al., 2015), *reliability* (Kobusińska and Hsu, 2018), and *ease of use* (Bilgihan et al., 2015). With respect to the lesser-valued characteristics, it is noteworthy that key characteristics for *service and system security*, so crucial to an virtual medium (Chang et al., 2014), have lost digital quality measurement value, probably on the basis of the company’s reputation for ethics and honesty (Caruana et al., 2015). In sum, and in corroboration of the importance of the relationship between quality and experience, it can be said that *system quality* provides the greatest customer satisfaction, that is, how the service has been implemented (load factor of 0.855), very appropriate in a channel in the process of consolidation and confirming the importance of technology in customer satisfaction with virtual service (Al Sokkar and Law, 2013; Michalco et al., 2015).
(4) Digital customer experience acts as a partial mediator on the model (the variance accounted for variable—VAF—of 40% ensures partial mediation, Hair et al., 2017), reinforcing the total effect of customer expectations on the satisfaction, due to the corresponding “wow” effect on the user. This conclusion is significant in the research framework, since the confirmation of expectations has traditionally been regarded as the determinant of satisfaction (**H2**) (Oliver, 2014). However, the full potential is demonstrated when the customer’s expectations can be surpassed (**H1**) (Michalco et al., 2015).
(5) The importance of expectations in the model. Pelegrín Borondo (2013) reflects on the type of expectation the user establishes compared to the experience they receive, from which we can infer that the change of sign of the expectations–experience effect may be due to an absence of the “wow” effect (factor 0.936), the projected ideal (Hall, 2012), whereby objectives are merely fulfilled, in a regulation or predictable way (factor 0.948) (Oliver and Burke, 1999). However, as stated by Michalco et al. (2015), it is common for customers to experience a distortion of their expectations (Huang et al., 2015) or of reality (Raita and Oulasvirta, 2011), from which we deduce that the difficulty faced by companies in managing the customer experience can only be solved with the proper information architecture (the most-valued characteristic), where what happens and what is expected are a single entity. The properly informed user will also be more sensitive to qualitative changes, meaning that companies must maintain speed, functionality, appeal, and challenge but manage the “wow” effect wisely in their private customer web areas to maintain the vibrance of use of the channel, essential in the new knowledge economy (Rodríguez-Ardura et al., 2018).

## Theoretical Implications and Management Implications

Key economic observers (Berger, 2017; Instituto Nacional de Estadística [INE], 2018a,b; iabSpain, 2018) augur a significant impact of digital transformation on countries’ economic growth in an imminent timeframe. As indicated by the DESI Index (Digital Economy and Society Index), the level of competitiveness of companies in the European Union evolves according to expectations and the digitization of data management (storage and sharing of open data, (Comisión Nacional del Mercado de Valores [CNMV], 2019), although it is true that it is very disparate according to economic sectors and countries (Paradigma Digital, Rodríguez Calvente, 2019).

However, there are serious indications that warn of the danger of turning digital transformation into a technological renewal rather than a new approach to customer encounter in market developments (It User, 2018). A redesign of the services according to the expectations of the clients is necessary (Congress DEC 2019).

The insurance field is a traditional sector that provides so-called experience products. The perception of the risk of this type of services means that the adoption of the channel was not relevant until the very recent past (Inese, 2018). Customers prefer face-to-face channels to ensure information and personal treatment by agents from whom they obtain security (Minsait, Asociación para el Desarrollo de la Experiencia de Cliente (DEC), 2019 report on digitization in Spain). The idea that “customers don’t just buy products or services; they also buy experiences and relationships” (Liferay Inc, 2019) is evidence, and competitive pressure and centricity vision (Kohli et al., 2019) impose a roadmap of no return in the digital transformation of the insurance sector. That is why it is considered relevant to present a contrasted behavior model that facilitates the implementation of the digital channel by the insurance sector, within the investigative prudence on the bias that the sample may present at the international level.

These results are more relevant than ever in the pandemic stage experienced in 2020, where society has faced unprecedented digitization and where only platforms where the customer has been taken into account and from impeccably implemented services have triumphed. According to the Capgemini Global Report (2020), customers are adopting a “millennial” mindset and increasingly relying on their own judgment based on information on the Internet and purchasing insurance products themselves. Thus, bigtech and alerted providers offer innovative and personalized products that offer a better customer experience (CX), within a competitive strategy, also unprecedented. That is why all new research is relevant as it provides knowledge to companies in their career for digital transformation and digital customer orientation.

Therefore, this article aims to highlight the relevance of the digital service as an added value for the customer. To do this, the article proposes to the scientific and insurance business community the model of customer experience, related to satisfaction and expectations and considers the following recommendations based on the results obtained. At the theoretical level, this article reinforces the theoretical framework on the customer experience, scarce in the reference literature that has usually focused on perceived satisfaction. The statistical robustness of the construct allows to consider its determinants as predictive of the customer experience. Although the variables of the model are a well-known benchmark in the field of multisectoral and omni-channel customer behavior research, the proposed model questions the relevance of expectations in the digital field of insurance and raises a new vision where it is established that it is the quality that determines a true customer experience in this context. Thus, the theory initiated by Davis (1989) and fully current to this day is questionable in the field of private insurance areas. That is, the idea that the user’s attitude toward technology conditions their use of it and that this attitude becomes an evaluative judgment on the digital channel (Worchel et al., 2003) can be solved by brands from an excellent digital quality and from co-creation with the user.

Therefore, it can be concluded that the model determines the true role of expectations, which, while important in the pre-digital phase, is the perceived quality that determines the true customer experience. On the other hand, surprising the client digitally is revealed as a guarantor of their satisfaction, as the predictive nature of both constructs is statistically confirmed. This is a remarkable discovery compared to the competition and the benefits it entails since it allows the “industrialization” of the customer experience, absolutely current in the new customer centricity vision.

Finally, and in the context of co-creation, the study provides an exhaustive study of quality based on the grouping of attributes based on its objective: platform, service performed, and information. This has made it possible to highlight the relevance of the information in the digital service as well as the perceived usefulness of the channel with respect to the service achieved. Knowing the digital attributes most valued by users allows the implementation of more ergonomic platforms open to joint innovation. That is, by creating an authentic digital experience, the adoption of the channel by customers is guaranteed, in which companies can establish a continuous and open dialog with them, which favors retention and recommendation to their influence groups.

On a practical level, insurance companies should monitor the implementation of their website, where it should encourage the use of the web channel to ensure training in the website, achieve an adequate web atmosphere that replaces the agent, look for the wow factor in the user that impact on recommendation, strengthen digital trust in channel usage, coordinate brand communities to achieve fidelity and recommendation, and invest in customer knowledge through “Digital Customer Voice” programs and know their needs.

Translated into concrete recommendations, insurance companies are advised to monitor and incentivize the following points in their digital strategy:

• The private area must be provided with a correct information architecture that facilitates the service autonomously, since it is one of the most valued quality attributes in risk products and even more so when they are provided from digital self-services.
• In the insurance sector, trust is the basis of the service. For this reason, extreme software quality strengthens trust and reliability. Ensure that transactions are carried out efficiently and securely and the continuous availability.
• Provide the perceived utility channel, implementing the most demanded and complete services from start to finish. Achieving the purpose of the service is one of the pillars of the digital customer experience.
• Know the customer, identify profiles, and listen to their needs through “Voice Of Customer Programs” so that it is possible to customize the services, according to their expectations. Know the customer, identify profiles, and listen to their needs through “Voice Of Customer Programs” so that it is possible to customize the services, according to their expectations.
• Incorporate the customer in the decision-making and co-creation processes of digital services. According to the latest report on global insurance from the Capgemini consultancy (2020), hyper-personalization is the key.
  Not only does it provide information and improve deployment but also it gives value to the customer and strengthens the relationship with the company.
• Dose the surprise effect, with continuous but adequate changes of the private customer web areas and always from the acquired knowledge of its users. Exceeding the expectations of the insurance user guarantees the customer experience and satisfaction.
• Provide complete services from an omni-channel strategy. Adapt the web strategy, so that the perception of the client is omni-channel and according to the reputation and brand image and innovation of the company.
• Improve the sense of community through proper management of customer areas from a progressive approach to new technologies. Digital expectations are not easy to standardize so they require a careful approach to them to encourage their adoption.

## Future Lines of Research and Limitations

The main limitation of the present study lies in the selected sample, i.e., insurance company customers. Subsequent research should seek to test the model in another industry with several different products and/or services. For the same reason, the selection of a single company may produce potential bias in the response, since the customer’s relationship with the company may distort the reality they perceive, as explained by cognitive dissonance theory. To overcome this limitation, we propose a study across many companies in different sectors.

Future studies should also include additional client profile variables, such as frequency of use (indicator of previous experience). This will enable a company to anticipate and manage the “wow” effect on an informed user, avoid any potential distortion of expectations and its corresponding impact on the customer’s experience, and shrewdly ration the (highly valued) information required throughout the process.

Finally, we wish to add that the digital customer experience continues to be analyzed in ideological or statistic–descriptive terms in companies, which makes it necessary to contribute to the creation of a new model. Moreover, the creation of new relationships is being driven by new digital quality characteristics oriented toward m-commerce, as well as by customers’ emotions (Boston Consulting Group, 2018) and how these affect their connection to a brand.

## Data Availability Statement

The raw data supporting the conclusions of this article will be made available by the authors, without undue reservation.

## Ethics Statement

Ethical review and approval was not required for the study on human participants in accordance with the local legislation and institutional requirements. The patients/participants provided their written informed consent to participate in this study.

## Author Contributions

All authors listed have made a substantial, direct and intellectual contribution to the work, and approved it for publication.

## Conflict of Interest

The authors declare that the research was conducted in the absence of any commercial or financial relationships that could be construed as a potential conflict of interest.
